# Layered defense: how mucus and tight junctions seal the intestinal barrier

**DOI:** 10.1007/s00109-017-1557-x

**Published:** 2017-07-13

**Authors:** Christopher T. Capaldo, Domonica N. Powell, Daniel Kalman

**Affiliations:** 1 Department of Cell Biology, Emory University School of Medicine, 615 Michael Street. Whitehead Research Building #143, Atlanta, GA 30322 USA; 2 Department of Pathology and Laboratory Medicine, Emory University School of Medicine, Atlanta, GA USA

**Keywords:** Mucus, Tight junction, Epithelial barrier, Inflammation, IBD

## Abstract

The colonic mucosa provides a vital defensive barrier separating the body from the microbial populations residing in the intestinal lumen. Indeed, growing evidence shows that loss of this barrier may cause disease or exacerbate disease progression. The loss of barrier integrity increases the translocation of bacterial antigens and stimulates inflammation in the intestinal mucosa, which is the central pathological feature of inflammatory bowel diseases (IBDs). This review focuses on how intestinal mucus and intercellular tight junctions (TJs) act together to maintain the integrity of the colonic barrier and how barrier integrity is dysregulated in IBD.

## Introduction

Inflammatory bowel diseases (IBD), encompassing Crohn’s disease (CD) and ulcerative colitis (UC), are characterized by chronic or intermittent inflammation of the intestinal mucosa [1]. IBD is an idiopathic and multifactorial condition, and recent studies indicate that both genetic and environmental factors contribute to the disease [2, 3]. During the etiology of IBD, aberrant immune responses triggered by the microbiota itself or excessive leakage of bacterial antigens into the mucosa cause inflammatory responses that progressively degrade the intestinal epithelia. This in turn permits more antigen leak and exacerbates inflammation, further compromising barrier integrity. The mucosal barrier integrity, therefore, is a key determinant of disease initiation, progression, and severity in IBD patients. Disrupting the inflammatory feedback cycle is a fundamental goal of IBD therapy. In experimental models, exposure of epithelial cells to proinflammatory cytokines such as tumor necrosis factor-α (TNF-α) causes cell death, alters the production of secreted mucins, and disrupts the epithelial barriers) [4–8]. Notably, the anti-TNF-α monoclonal antibody, infliximab, a treatment for advanced IBD, short-circuits this inflammatory feedback loop by limiting immune activation and mitigating cytotoxic effects [5].

**Fig. 1:**
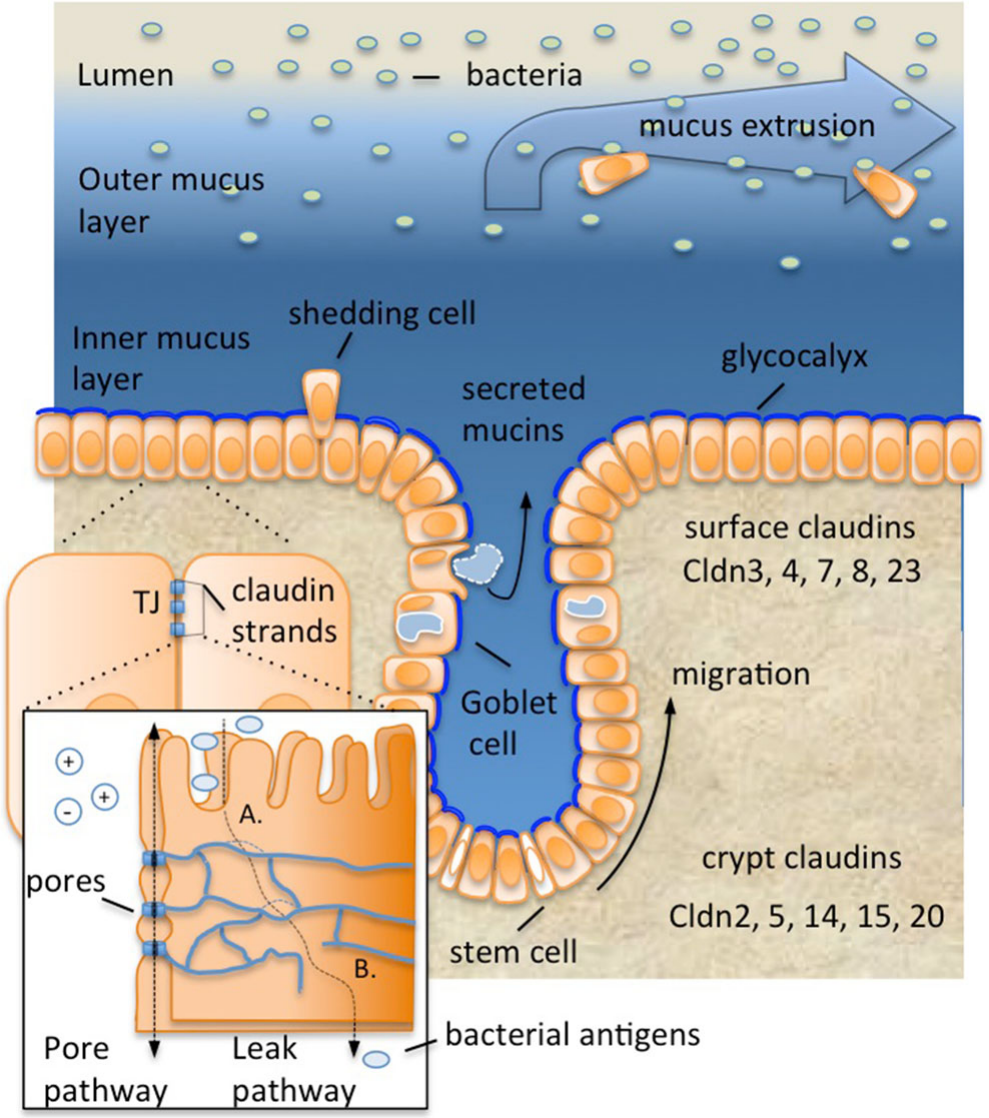
Protective layers of the colonic mucosal barrier. The outer mucus layer, composed of Muc2 and various carbohydrate modifications, interacts with colonic microflora, while the density of the inner layer prevents bacterial penetration. The innermost mucus layer, the glycocalyx, is attached to the plasma membrane. Together, the inner and outer mucus layer limit abrasion and trap bacteria, thereby restricting their contact with the epithelia. In general, the mucus limits contact of bacteria with underlying epithelial cells but does not restrict access of bacterial metabolites. The epithelial paracellular barrier is composed of intercellular contacts called tight junctions (TJs). TJ strands, composed of proteins called claudins, connect apposing cells and occlude the paracellular space. Some claudins form ion pores within the TJ (pore pathway, *inset*), which selectively permit ion and water exchange. However, bacterial products may breach TJ defenses upon separation (**a**) or rupture of the TJ strands (**b**). Epithelial cell turnover likely helps to remove attached bacteria, and mucus flux ensures trapped bacteria are eliminated. Stem cells residing in the crypt bases produce progeny that migrate toward the lumen surface. While doing so they differentiate, producing goblet cells that secrete mucins. Differentiating cells also change the complement of claudins that they express, such that pore forming claudins are more highly expressed in the crypt-base compared to the luminal surface [54]

Epithelial tissue acts not only to separate essential internal organ systems from the outside world but also facilitates communication with the microbiota and absorbs nutrients. Additional features of the colonic barrier include secreted mucus and water absorption. The epithelial layer is composed of a simple monolayer of around 20 billion contiguous cells [9], which, like the skin, continually regenerates. Stem cells, located at the base of the crypts of Lieberkühn, produce daughter cells that differentiate. As the progeny cells migrate toward the luminal surface, they lose their proliferative capacity and differentiate into specialized cells, which include colonocytes and mucin producing goblet cells (Fig. 1) [10, 11]. Cells at the luminal surface eventually undergo programmed cell death (apoptosis) and are shed (Fig. 1). From birth to death, the regenerative process is complete within a few days [12].

Mature cells facing the lumen, termed surface cells, are in close apposition to layers of mucus. The cells and the mucus together form a layered barrier separating the body from the colonic microflora and limiting influx of bacteria and bacterial antigens. The combination of the epithelial sheet and the mucus layers forms a biologically flexible and environmentally responsive barrier to luminal contents. Indeed, growing evidence shows that loss of barrier integrity may cause disease or exacerbate disease progression (reviewed in [13, 14]). This review focuses on how the mucus and TJs together provide a layered defensive barrier in the colon and how their dysregulation contributes to IBD.

## Mucins separate the epithelia from the intestinal lumen

The colonic mucosa secretes copious amounts of mucus, which is composed of complex and extensive O-linked oligosaccharide modifications on a mucin protein backbone. These glycoproteins form large disulfide-linked macromolecules, and upon release from goblet cell granules into the lumen, become hydrated and expand to form a net-like gel [15]. Whereas the small intestine contains a single layer of loose unattached mucus, mucus in the colon is organized into three distinct layers. Membrane-anchored mucins associated with the colonic epithelial cells form the glycocalyx. The glycocalyx gives way to a second tightly crosslinked inner layer primarily composed of the mucin protein MUC2. The outermost layer, generated by proteolysis of the inner layer, is less dense and less viscous. A more detailed description of mucin organization in the colon is reviewed elsewhere [16–18].

These mucus layers serve to separate bacteria in the lumen from the epithelia in several ways. First, the mucus layers achieve different densities, such that commensal flora or even pathogens can reside within the low density outer layer [16, 18] but are generally excluded from the high density inner layer and glycocalyx [19]. The outer mucus layers contain diverse carbohydrate motifs, immunoglobulins, and other proteins that serve as binding sites for bacteria [20, 21]. A second means of separating the bacteria is mucus turnover. As secreted mucus is extruded, proteolyzed in the inner layer, and ultimately secreted into the lumen, bound bacteria are expelled and removed from the body by intestinal peristalsis. The mucus layers turn over in a matter of hours, providing dynamic removal of bacteria and limiting their access to the epithelium [22]. Finally, the mucus serves as a lubricant to prevent feces from abrading and tearing the epithelia [22]. Thus, the mucus serves to limit access of bacteria to the epithelia. It is important to note that the density of the mucus does not preclude access of secreted bacterial metabolites or toxins to the epithelia based on size alone. Given the importance of these molecules in epithelial barrier integrity, a role for the mucus in regulating their access promises to be an increasingly important area of investigation.

Defective mucin production or processing has been linked to human IBD. UC patients have fewer goblet cells and decreased synthesis and secretion of MUC2, especially during episodes of severe disease. This allows direct contact of the colonic microbiota with the epithelial barrier [23]. In addition, lower levels of the goblet cell differentiation factors HATH1 and KLF4 were evident in biopsies from patients with active UC [24]. Furthermore, genome-wide association studies (GWAS) have implicated mutations in the *MUC* genes in IBD pathogenesis, including the membrane-anchored MUC3 and MUC19 [2, 25]. Moreover, variants of MUC2, the major secreted mucin in the human colon, have been found in IBD cases [26].

Unlike UC patients, CD patients exhibit *increased* mucus production [24, 27]. However, despite the increased levels, the mucus barrier still fails to restrict bacterial access to the epithelia [28]. These observations suggest that secondary structural modifications, in addition to mucin levels, are critical for function of the mucus barrier. In this regard, it has been proposed that increased bacterial access is due to impaired mucin processing in CD patients, which may affect the length of glycans attached by O-glycosylation [8, 23]. Notably, proper glycosylation, sulfation, and sialylation are essential for the viscoelastic functions of mucus. Humans and mice with IBD have been found to have higher levels of sulfide, a product of sulfate reducing bacteria, that could reduce the disulfide bond between mucins and degrade the mucus network, thus allowing increased microbial contact with the host [29, 30]. Accordingly, glycosylation and sulfation defects have also been found in a UC cohorts, indicating that mucus modification may play a role in limiting barrier function in disease [31–33]. More specifically, mutations in core 1 β3GalT-specific molecular chaperone (*Cosmc*), a chaperone for the T-synthase glycosyltransferase responsible for the synthesis of the O-glycans on mucin proteins, have also been associated with IBD in GWAS studies [34]. The location of *Cosmc* on X chromosome may provide an explanation for the male gender bias of IBD [35].

Experimental disease models in mice further strengthen the role the mucus barrier and mucins can play in the prevention or pathogenesis of IBD. For example, 5 weeks after birth, Muc2 knockout animals (Muc2^*−/−*^) develop spontaneous colitis and display increased susceptibility to experimental DSS colitis, presumably due to the direct contact of intestinal microbiota with the epithelia [36]. Animal models also demonstrate that glycoprotein modification is crucial to intestinal homeostasis. Mice lacking core 1-derived O-glycans, recapitulating defects in humans with mutations in Cosmc, show loss of mucus complexity and rapid spontaneous colitis [31]. It is important to note that the outer and inner mucus layers of the colon are almost entirely composed of Muc2, and it is expected that Muc2^−/−^ strains would then depend only on the glycocalyx for fecal lubrication [21]. In summary, the secreted mucus barrier appears to function as a means of preventing or limiting contact of bacteria and bacterial antigens with epithelial cells.

The outer layer of colonic mucus is a habitat for both indigenous and transient microorganisms, called autochthonous and allochthonous, respectively. Changes in the resident autochthonous bacteria appear to have more impact on the host’s health than do changes in transient luminal allochthonous bacteria found in the fecal matter [37]. An imbalance of the microbiota, a state referred to as dysbiosis, is characteristic of IBD, indicating the importance of maintaining the appropriate bacteria in the intestinal mucus [38]. Mucus provides nutrients to bacteria, including amino acids and sugars, which are especially important for those bacteria capable of degrading the glycans on the mucin backbone [39, 40]. *Akkermansia muciniphila* is a mucus-degrading bacterium underrepresented in many disease states including IBD, obesity, and type 2 diabetes, and numerous studies have correlated the presence of *A. muciniphila* with a healthy mucosa [41, 42]. Additionally, providing mice with *A. muciniphila* during high-fat diet (HFD) feeding, which normally results in decreased barrier integrity, led to a restored barrier, increased goblet cell numbers, and prevention of metabolic endotoxemia [42] [43] [44]. Interestingly, bacteriophages within the mucus layer may also dictate the abundance and diversity of bacteria found in the intestine [38]. One study of an IBD cohort demonstrated an inverse correlation between bacteriophage expansion and diversification on the one hand and bacterial diversity on the other, raising the possibility that bacteriophages may contribute to the dysbiotic state in IBD [45]. Taken together, these studies demonstrate the importance of intestinal mucus in supporting growth of protective commensal bacteria as well as facilitating repopulation and maintaining commensal homeostasis to prevent dysbiosis. Accordingly, disruption of the mucus barrier may result in dysbiosis.

## TJs form a paracellular seal

Epithelial cells are networked together through proteinaceous adhesive contacts called junctions, which both join cells together and provide a paracellular seal. The seal between cells requires tight junctions (TJs), a specialized multipurpose adhesion that simultaneously occludes the paracellular space, dictates ion flux across the tissue, and maintains cellular polarity. The TJs are positioned at the boundary of the apical and lateral membrane surfaces of adjacent epithelial cells in the colon and consist of 5–7 membrane fusion sites called “kissing points” [46, 47]. The entire circumference of each cell is joined to apposing cells via an adhesive TJ band, called a strand. TJ strands in the colon are not linear but rather highly branched structures that form anastomosed webs that extend several hundred nanometers laterally from the apex of the cell. All epithelial cells that line the intestine are joined in this manner.

TJs regulate the flux of ions and solutes on the one hand, termed the “gate function,” and maintain cell polarity on the other, termed the “fence function” [48, 49]. Thus, TJs serve as a barrier to bacteria and bacterial products while also corralling apical plasma membrane proteins, and presumably the glycocalyx mucins, at the lumen-facing domain of the epithelial cell.

The claudin family proteins are essential components of TJs. Claudins form TJ strands by polymerizing within the plasma membrane and dimerizing with claudins on apposing cells, across the extracellular space, to generate the paracellular seal. There are 24 claudin genes in humans, with multiple claudins expressed within any given cell [50]. Importantly, several claudin proteins dimerize to form charge and size-selective ion pores that are vital for ionic homeostasis in epithelial tissues. For example, mice deficient in both claudin 2 and 15 mice fail to equilibrate sodium levels in the luminal space of the small bowel, which leads to low nutrient absorption, wasting disease, and premature death [51]. Other claudins, such as claudin 4, appear to promote a tighter seal; claudin 4 does not form ion pores within the TJ but appears to exclude pore-forming claudin 2 [52]. The permeability of the TJ is thought to derive at least in part from the relative amounts of amounts of pore forming or sealing claudins within the stands, as well as the architecture of the strands, particularly the complexity and numbers of TJ strands [49, 53].

Different complements of claudins are expressed at different levels in epithelia along the length of the intestinal tract, as well as within the intestinal crypts themselves [52] (Fig. 1). For example, our recent studies in mice indicate that 11 claudins are expressed as a gradient within the crypts (Fig. 1) [54]. In general, “leaky” pore forming claudins are restricted to the colonic crypt base, whereas “tight” sealing claudins are more prominently expressed in surface cells facing the lumen.

At the molecular level, the TJ is a highly diverse structure composed of both transmembrane and cytoplasmic proteins [55, 56]. Besides claudins, there exist three additional classes of transmembrane proteins in the TJ: occludin, tricellulin, and junctional adhesion molecules (JAMs) [53, 57–59]. A dense “plaque” of scaffolding molecules is anchored to the transmembrane proteins, which include the Zonula Occludins (ZO) and MAGUK family proteins (reviewed in [49]). These scaffolding proteins link the transmembrane proteins to kinases and signaling molecules that localize at the junction. These molecules in turn control not only cell-cell contacts but also the actin polymerization machinery and contractility apparatus of apically situated actin and myosin [49, 60]. Scaffold proteins have different affinities for claudins and may regulate the types of claudins in the TJs [61]. Likewise, the contractile machinery appears to regulate localization of claudins within the TJs [62–65]. In summary, this molecular signaling apparatus controls claudin localization and function, and thus the permeability of the epithelial barrier.

In addition to forming ion pores, claudin strands have a poorly understood mechanism that permits small molecules to traverse the TJ, termed the paracellular “leak pathway” [66–68]. There is accumulating evidence that TJ strands themselves are dynamic and frequently break, reform, and exchange claudin proteins in response to physiological, environmental, and pathogenic stimuli [52, 63]. However, it remains to be established whether the paracellular leakage results from separation of transcellular dimers, strand breakage, or some other unknown mechanism. Interestingly, several pathogens, including both bacteria and viruses, have evolved means to traverse the paracellular junctions by disrupting claudins, or the actin structures that provide structural integrity to the cell and the TJs (reviewed in [69]). For example, the bacterium *Clostridium perfringes* secretes a toxin that binds claudins 3 and 4, whereas Hepatitis C virus interacts with claudin 1 [69]. In summary, the composition and numbers of the TJ strands, the type of claudins that compose them, their localization within the intestinal tract, and the intracellular signaling apparatus all contribute to the permeability of the intestinal barrier.

Several lines of evidence implicate dysregulation of the mucosal barrier, and of TJ architecture and claudin expression in particular, in the etiology of IBD [47, 70]. GWAS studies have identified several genes that link TJ function to IBD [2, 71]. Of the IBD implicated genes, one of the best understood is hepatocyte nuclear factor alpha (HNF4a) [72]. HNF4a is a transcription factor involved in the maturation of colonocytes as they migrate out of the crypts. HNF4a regulates claudin expression, including claudin 7 [73]. Moreover, multiple studies have demonstrated a change in TJ transmembrane proteins in IBD patients. Tricellulin, a specialized occludin-like molecule responsible for sealing the TJ at tricellular contacts, is decreased in UC [74, 75]. Moreover, expression of the sealing claudins 1 and 4 is suppressed in IBD patients [70, 76]. Furthermore, upregulation of claudin 2 expression and downregulation of claudin 5 and 8 correlate with barrier dysfunction and active CD [77]. A more comprehensive review of the TJ and TJ-associated proteins dysregulated in IBD can be found elsewhere [75, 78, 79].

Disease phenotypes of human IBD are recapitulated in mice with genetic deficiencies similar to those found in human patients and confirm the importance of altering TJs and barrier integrity in IBD pathogenesis. For example, mice deficient in claudin 7 in the colon develop lethal colitis soon after birth [80, 81]. Notably, based on freeze fracture EM analysis, TJs of wild type and claudin 7 knockout animals have nearly identical structure, yet the character and function of the TJ appears compromised. Without claudin 7, the epithelial barrier is more permeant to small molecules (~400 Da), although the flux of larger molecules (~4 kDa) and the overall balance of Na^+^ and Cl^−^ were unchanged [80]. Therefore, claudin 7 appears to function to selectively regulate permeability of small molecules, and its dyregulation is sufficient to cause disease. The observation that loss of claudin 7 disrupts the TJ seal raised the possibility that this claudin might also limit flux of bacterial antigens. Accordingly, antibiotics ameliorated colitis in mice with claudin 7 deficiency, whereas addition of bacterial antigen (fMLF) reversed this protective effect [80]. Together, these data support a model of IBD pathogenesis in which dysregulation of TJs facilitates leakage of luminal antigens across the epithelial barrier that trigger inflammation and initiate colitis. Master regulators of claudin expression have also been implicated in mouse models of IBD. As in humans, Hnf4a regulates expression of claudin genes in mice [82, 83]. Accordingly, loss of function alleles of Hnf4a in mice results in dysregulation of claudin expression and spontaneous colitis [84].

## The mucosal barrier, inflammation, and IBD

The mucosal barrier is not a static structure, and epithelial tight junctions and mucus production both respond to inflammatory stimuli. For example, upon infection, the inflammatory cytokine TNF-α increases epithelial permeability through alterations in TJ function, structure, and dynamics [52, 85]. Yet, TNF-α also increases mucus production by goblet cells to limit the inflammatory response by stemming influx of bacteria through the mucus layers.

Importantly, sustained inflammation or protracted dysregulation of barrier integrity initiates or exacerbates IBD. In this regard, loss or dysregulation of either the mucus or the TJs suffice to cause colitis, an effect that depends upon bacterial or antigen translocation. Thus, mice lacking claudin 7, Hnf4a, and Muc2 all develop spontaneous colitis [72, 80, 86]. The observation that symptoms are relieved under germ-free conditions or after treatment with antibiotics highlights the non-redundant role of the mucus as well as TJs in limiting access of microbes and microbial products to the body and the contribution of bacterial antigens to colitis. Additionally, the mucus barrier and TJs are interdependent such that loss of one diminishes the other. In this regard, Muc2^−/−^ mice display increased epithelia barrier permeability and dysregulated claudin gene expression in addition to defects in the mucus [86]. Likewise, numbers of mucin-producing goblet cells and mucus are diminished in Hnf4a^−/−^ mice [84]. Such interdependence could result from dysregulation of signals that coordinate both the mucus and TJs. Alternatively, inflammation associated with either knockout phenotype could damage the remaining barrier. In any case, such interdependence may facilitate feedback inflammatory responses that perpetuate IBD.

## Summary and future directions

The epithelial TJs and the three mucus layers cooperate to form a highly integrated barrier system that together limit access of luminal contents to the body proper. The molecular constituents of this barrier are still being identified and their functions elucidated. Nevertheless, through in vivo and in vitro studies in experimental models, as well as studies of human IBD, an integrated understanding of mucosal barrier function is beginning to emerge. The capacity of the mucus to prevent abrasion and trap bacteria represents the first line of defense, while the paracellular TJ barrier prevents leakage of bacterial antigens from the lumen into the body. Furthermore, the rapid turnover of both mucus and lumen-facing epithelial cells ensures that bacteria that do interact with these structures are constantly being evicted. This multilayer system thus functions to limit bacterial contact with the host while still permitting access of small molecules, including microbial metabolites.

A comprehensive understanding of the epithelial barrier system and its relationship to commensal microbes and to the immune system may provide a more integrated approach to treatment of IBDs. For example, strategies aimed at strengthening both the mucus and epithelia barriers and thereby reducing exposure to inflammatory antigens, coupled with therapeutics that reduce susceptibility of the mucosa to damage caused by bacteria or by inflammatory responses, may restore the intestinal tract of IBD patients to a more benevolent state.
